# Description and validation of a new, simple, easy-to handle, point-of-care technique for measuring erythrocyte aggregation kinetics

**DOI:** 10.1038/s41598-022-18410-6

**Published:** 2022-08-30

**Authors:** Olivier L. Charansonney, Pascal Morel, Jacques Dufaux, Eric Vicaut

**Affiliations:** 1 Cardiology Department, Centre Hospitalier Sud-Francilien (CHSF), Corbeil-Essonnes, France; 2 Clinical Physiology Department, Hôpital Lariboisière, Paris, France; 3 Etablissement Français du Sang (EFS), La Plaine Saint Denis, France; 4 UMR 1098 INSERM, Université de Franche-Comté, Besançon, France; 5 University of Paris, Paris, France; 6 Clinical Research Unit, Hôpital Fernand-Widal, Paris, France

**Keywords:** Diagnostic markers, Blood flow

## Abstract

Erythrocyte aggregation (EA) is a physiological process by which erythrocytes reversibly stick together within the blood vessels. EA plays a major role in blood viscosity in vivo, thereby impacting blood flow to organs. EA is no doubt greatly important in both physiological and pathophysiological conditions, but the studies its importance calls for are complicated by the lack of a reliable and easy way to measure it. We have developed a new point-of-care technique which can very specifically measure EA initial kinetics (EAK) in 20 s directly on blood samples routinely collected in tubes commonly used in clinical settings. We present the results of the validation studies of this EAK test: A mono-exponential curve explains 99% of EAK variance. EAK is normally distributed in healthy individuals, with an interindividual 15% coefficient of variation and is stable for least one hour after blood collection. Intraindividual coefficient of variation is 2.6%. EA can now be easily measured in any clinical setting.

## Introduction

Erythrocyte aggregation (EA) is a reversible process which takes place in low shear stress conditions such as venous circulation. In arterial microvessels it also leads to a decrease in blood viscosity: the Fahraeus-Lindqvist effect^1^. For example, we were able to show that the magnitude of EA can impact coronary blood flow. A physiological degree of EA increases the flow beyond both non- and hyper- aggregation^2^. Increased EA in many disease conditions has been well documented^3,4^ and is of specific interest for diseases with a strong inflammatory component. Some diseases with acute inflammation need a fast diagnosis and treatment: severe infections, arteritis (temporal arteritis…), acute coronary syndrome or acute stroke. Other diseases with low-grade chronic inflammation such as type 2 diabetes and rheumatic arthritis may also benefit from a specific evaluation of EA.

EA was historically assessed by measuring the erythrocyte sedimentation rate (ESR) calculated over one or two hours. ESR is dependent on EA, at least for its initial phase (first minutes)^5^; however, ESR is a long term consequence of EA. ESR remains a marker of inflammation in clinical practice. It can now be measured with automated analyzers in less than a minute (TEST-1)^6^.

The physiological and pathophysiological impacts of EA on circulation have been studied for a century. Since the 1980s erythrocyte aggregation has been intensively studied with new instruments that can analyze aggregation during the first 10–30 s. The technique most commonly used over the past 40 years requires pouring the blood sample into a measuring chamber such as a Couette viscometer cylinder or any kind of chamber which can enable an optical measurement of EA^7^. Indexes describing the EA kinetics, such as half-lives and areas under the curve, are integrated over the first 10 s of the process and beyond^8^. This technique needs blood manipulation, is time consuming, and has relatively high variability^4,7,8^. New commercially available analyzers (RheoScan D), based on microfluidic technique, can measure erythrocyte aggregation in less than two minutes. EA is measured by light transmission, and critical shear stress (CSS) is measured by light backscattering. This technique has been used in clinical settings, mainly in patients with diabetes and vascular diseases^9^. All the commercially available analyzers (TEST-1, Rheoscan) need a blood sample taken from the blood collection tube. Another technique using quantitative ultrasound might be used in vivo^4^. However, this technique has not been validated yet and requires a robust gold standard for validation.

The techniques currently used are not designed to study the early phase of EA, in which the first interactions between single erythrocytes could have a very significant impact on blood flow in arterial microvessels where the Fahraeus-Lindqvist effect takes place.

Furthermore, none of these techniques can be used at the point-of-care (e.g. on an emergency trolley or in a mobile unit). This limit precludes the use of EA for assisting clinical diagnoses and decisions in emergency settings.

We designed a specific point-of-care device to explore the initial phase of the aggregation kinetics (EAK) and validated its characteristics with clinical studies in both healthy individuals and patients.

## Results

### Study A: healthy subjects

Fifty-one healthy donors (32 women and 19 men) were included in Study A. The mean age was 26.5 years [19–56]. For every subject EAK was very accurately described by a mono-exponential curve. The first 5 s segment of the kinetics was fitted and the half-life, later referred to as EAK5s, was computed. The fitting coefficient r^2^ was equal to or greater than 99% in all samples analyzed. The mean EAK5s was 2.51 ± 0.38 s. The interindividual coefficient of variation was 15.0%. No difference was observed between the 32 women and the 19 men (*p* value 0.10). EAK5s was normally distributed in healthy subjects: *p* value for normality is 0.32.

We tested Model 1 (linear fitting) and Model 2 (mono-exponential fitting) on the EAK5s data obtained from 18 blood samplings (17 subjects). For Model 1 the mean and median fitting coefficients r^2^ were both 0.96. For Model 2 the mean and median coefficients were both 0.998. Therefore, Model 1 explained 96% of the variance of the experimental data, and Model 2 explained 99,8% of the variance. The initial EAK kinetics follows a mono-exponential model and the only limiting factor is the aggregation process, not the concentration of isolated erythrocytes.

We measured EAK5s 30 times in two different tubes filled with the same blood. The coefficients of variation for the two sets of measurements were 2.3% and 2.4%. There was a very small (0.2%), statistically insignificant difference between the mean EAK5s of the two sets of measurements. So, EAK measured with our technique is not significantly influenced by the optical characteristics of the tube, at least not with the type of tube used in our studies.

### Study B: patients with cardiovascular conditions—comparison with healthy subjects

**Figure 1:**
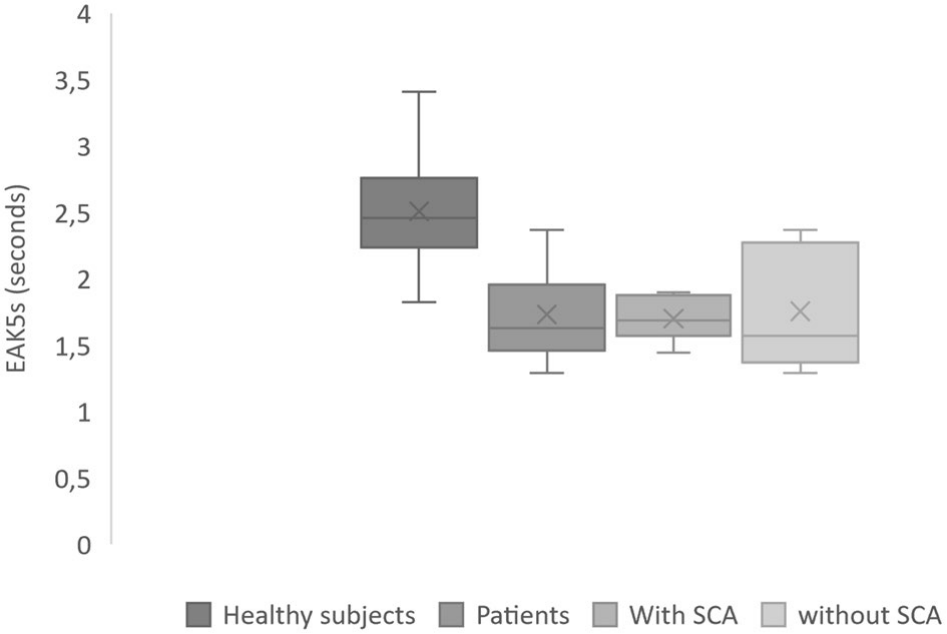
Box-plot of EAK5s in healthy subjects and patients with and without acute coronary syndrome (ACS).

We compared results from patients hospitalized in Centre Hospitalier Sud-Francilien’s intensive care cardiology unit with results from healthy subjects (Table 1 and Fig. 1).

**Table 1:** Main results Study B.

	Healthy donors	Patients hospitalized in cardiology	Patients with recent ACS	Patients without ACS
Number of subjects	51	14	6	8
Women (%)	32 (62.7%)	6 (43%)	2 (33%)	4 (50%)
Age (year): mean [range]	27 [20–54]	73 [38–94]	72 [38–92]	75 [61–94]
EAK5s mean ± SD (s)	2.51 ± 0.38	1.73 ± 0.34	1.70 ± 0.17	1.76 ± 0.44
Student t test Patients/healthy donors		< 0.0001	< 0.0001	< 0.0001
Coefficient of variation	15%	20%	10%	25%

*ACS* acute coronary syndrome.

The fitting coefficient r^2^ of EAK5s was equal to or greater than 99% for 9 of the patients. In the remaining patients, the best fit was computed from the shortest (1.5 s) segment in 4 patients (2 with ACS, two with other inflammatory conditions). One patient had the best fit computed from the 3 s segment. The fitting coefficients for the 10 s segment were equal to or greater than 99% in only 4 patients. Therefore, the fitting of a 10 s segment was not appropriate when analyzing patients’ blood. It justifies our choice of selecting EAK5s for our main analyses.

In any case, a mono-exponential model can fit the data with r^2^ ≥ 99% confirming our mechanical hypothesis in patients.

Patients had very significantly accelerated EAK5s compared with those of healthy individuals. However, in our study, patients were much older and more often men than were healthy individuals.

### Study C

Repeated measures were systematically taken the hour after blood collection from 10 patients visiting the Lariboisière emergency department. Nineteen to 22 measurements were performed per patient totaling 208 measurements. The 5 s segment model explained 99% of the variance in all measures. When considering EAK5s, mean and median coefficients of variation were 2.6% and 2.4% respectively. Measurements after 1 h were not different from the first measurements (*p* = 0.96). We measured both hematocrit and EAK5s in 138 patients. Hematocrit values ranged from 23.6 to 55.3%. The linear correlation coefficient between hematocrit and EAK5s was 0.26, excluding a strong effect of hematocrit on EAK5s. This is consistent with the mono-exponential model (Model 2) in which the time constant doesn’t depend on the concentration of isolated erythrocytes.

## Discussion

For the first time we have a technique which can measure erythrocyte aggregation in whole blood, at the point-of-care in less than a minute, using only standard-care blood collection in widely available tubes. Our results have shown that both in healthy individuals and in patients EAK initially follows a mono-exponential model which, as the very high coefficients of correlation (> 99%) prove, explains 99% of the variance. This is fully in accord with a two-by-two aggregation process predicted by Model 2. Therefore, our technique very precisely measures EAK. It depends only on the kinetics of backscattered light, not on the absolute values. This prevents our results from being influenced by the variations of the optical characteristics of the tubes. Furthermore, our results are not influenced by hematocrit (in accord with the model used). This contradicts the common belief that erythrocyte aggregation may be modified by hematocrit. However, our technique only explores the very beginning of the aggregation process (two-by-two aggregation). It is plausible that the formations of larger rouleaux and of tridimensional structures depend on hematocrit.

The intraindividual and interindividual coefficients of variation (2.6% and 15% in healthy individuals) are far less than those reported from commercially available devices (4.2–4.6% and 36–39% respectively)^8^. These commercial devices measure the aggregation process during 10–20 s. During this period, rouleaux can form in large number, especially in blood taken from patients with inflammation. It is therefore difficult to directly compare our results with those obtained with commercially available devices.

There are several limitations to our study:

- The number of healthy subjects was limited, and these subjects were young. Therefore, we have little information on the physiological variation of EAK, especially related to aging.
- The comparison between young healthy subjects and older, more often male, patients hospitalized in cardiology should only be considered as an indication that pathophysiological conditions may considerably accelerate EAK.
- We don’t know whether EAK is similar in venous blood (where stasis and thrombosis can occur) and in arterial blood (where the Fahraeus-Lindqvist effect takes place).

Despite these limitations, which could easily be corrected for in further studies, our technique enables extensive investigations of an (up-to-now) poorly understood physiological phenomenon: erythrocyte aggregation. Furthermore, these investigations, and the possibility of using the EAK test for medical diagnosis, can be done in any clinical setting at a very low cost: the device is built with low-cost on-shelf available components, uses a standard blood collection tube, requires no reagent, and there is no degradation of the blood sample, which can then be used for other measurements. The easy handling of the test needs no special training. The EAK test has only a marginal environmental impact.

## Materials and methods

The following descriptions of the experimental design first include the description of the device, then the design of the validating studies.

### Blood collection

Blood samples were collected by venipuncture at the point-of-care in regular blood collection tubes containing anticoagulant (a standard EDTA tube used for blood counts). These tubes are manufactured for a sampling volume of 4 ml. The tubes containing the whole blood were used for EA measurement without any other manipulation (dilution, sampling…).

### Device for EA measurement

#### Stirring techniques

Before measuring EAK, the blood needs to be stirred to disrupt all erythrocyte aggregates without altering the blood components (cells and proteins).

The tube containing the blood was inserted in the mobile optical part of the device.

**Figure 2:**
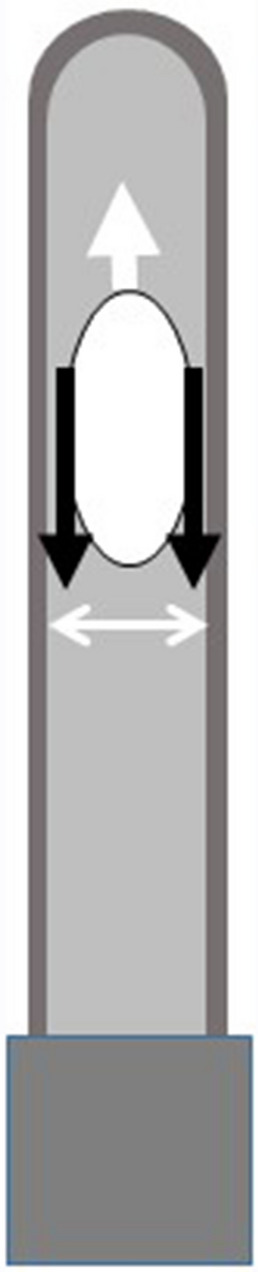
shear rate generated by the upside-down stirring technique: the air bubble moves upward (vertical white arrow) forcing the blood to move downward. The shear rate was computed from the tube internal diameter (white double-headed arrow), the horizontal diameter of the bubble (black double-headed arrow), the volume of blood and the period of the stirring motion (1 Hz).

The device automatically applied a 180° rotation movement to the optical part on an axis perpendicular to the tube’s longitudinal axis at its center of gravity (upside-down) for 10 s. This low frequency (1 Hz) stirring movement is similar to what the technicians or nurses are instructed to apply to the tubes immediately after blood collection by venipuncture (8–10 inversions of the tube). With this motion, the air bubble within the tube moves back and forth along the great axis of the tube (white arrow in Fig. 2). The shear rate is generated by forcing the blood to flow between the bubble and the tube wall. We measured the distance between the air bubble and the internal wall of the tube by taking a series of pictures of the tube during the stirring movement. Based on the tube’s geometry the shear rate was estimated at 1000 s^−1^ for a 1 Hz motion (shear rate equals velocity divided by the distance between the bubble and the tube wall). It is as high as the highest shear rates generated by commercially available devices. The shear stress, product of the shear rate and the viscosity, should be able to disrupt all erythrocyte aggregates^10^, even when the required shear stress (critical shear stress: CCS) is high. No foam layer is formed in the tube. The blood flow is laminar.

The blood sample can then be used for other laboratory measurements.

#### Optical measurement of EA

With most of the commercially available devices EA, was measured by illuminating a vessel containing the blood. In devices based on a Couette viscometer the external cylinder of the viscometer is transparent and the EA process is measured by analyzing the backscattering of incident laser light.

**Figure 3:**
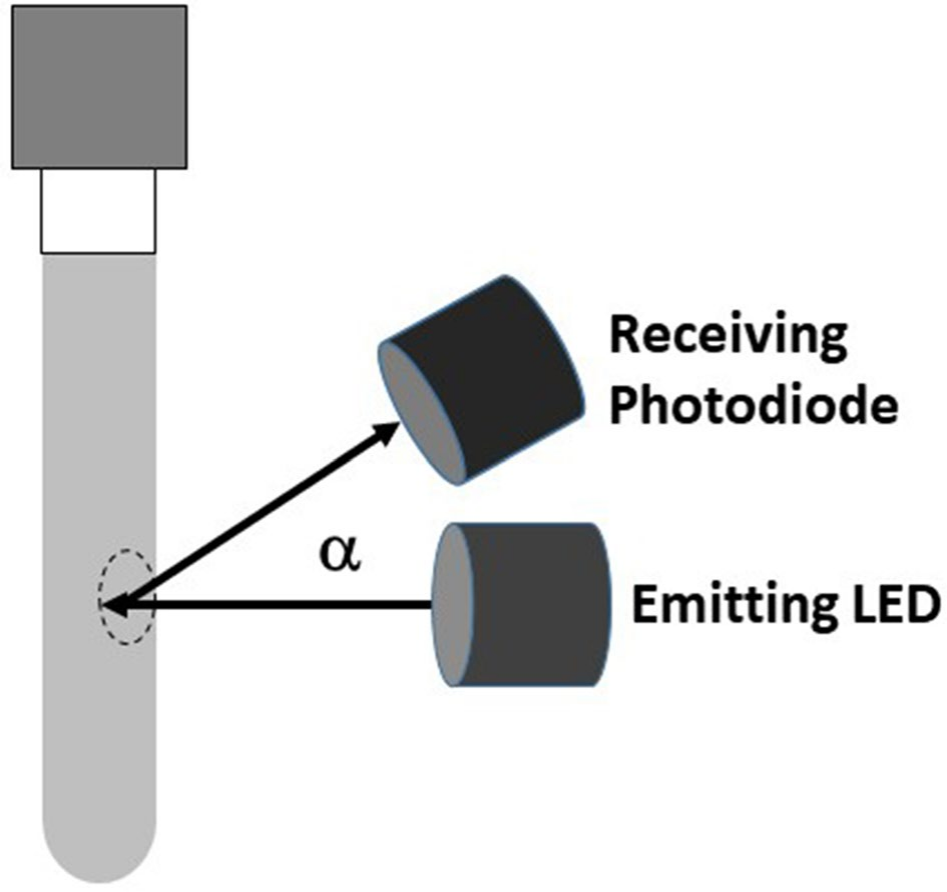
Measure of the light backscattered by a small blood sample (dotted circle).

For our device the stirring movement was applied to the optical part for 10 s. Then, the tube was illuminated by a perpendicular LED or laser source. The light backscattered by the blood was measured by a receiving photodiode positioned in the same plane at an angle α above the perpendicular (Fig. 3).

### Mechanistic hypothesis concerning early EA Kinetics

**Figure 4:**
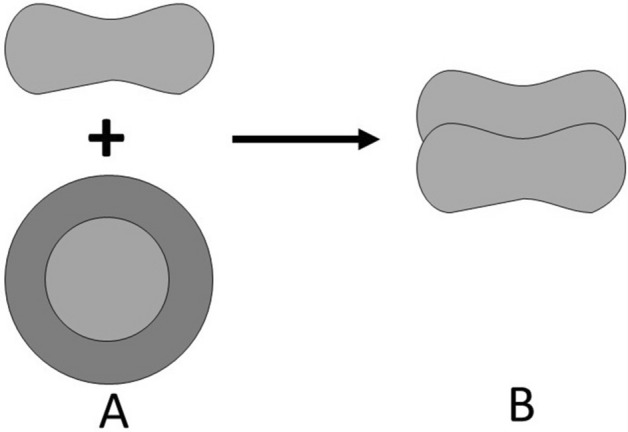
Two-by-two aggregation process.

The initial EA kinetics (EAK) consists of the formation of two-by-two aggregates of erythrocytes as shown in Fig. 4. Two models can describe this kinetics:

*In Model 1* the formation of two-by-two aggregates depends on the concentration of isolated erythrocytes: [A] + [A] → [B], giving the equation:

$$ \frac{d\left[ B \right]}{{dt}} = k \left[ A \right]x\left[ A \right] $$

where [A] is the concentration of isolated erythrocytes, [B] the concentration of two-by-two aggregates, and k the time constant of the kinetics. The solution is:

$$ \frac{1}{\left[ A \right]} = kt + \frac{1}{{{\text{A}}0}} $$

where A0 is the initial concentration of A forms.

*In Model 2*, A forms are very concentrated, and the limiting factor is the aggregation process itself: [A] → [B] giving the equation:

$$ \left[ A \right] = {\text{A}}0{ }\,e^{ - kx} $$

**Figure 5:**
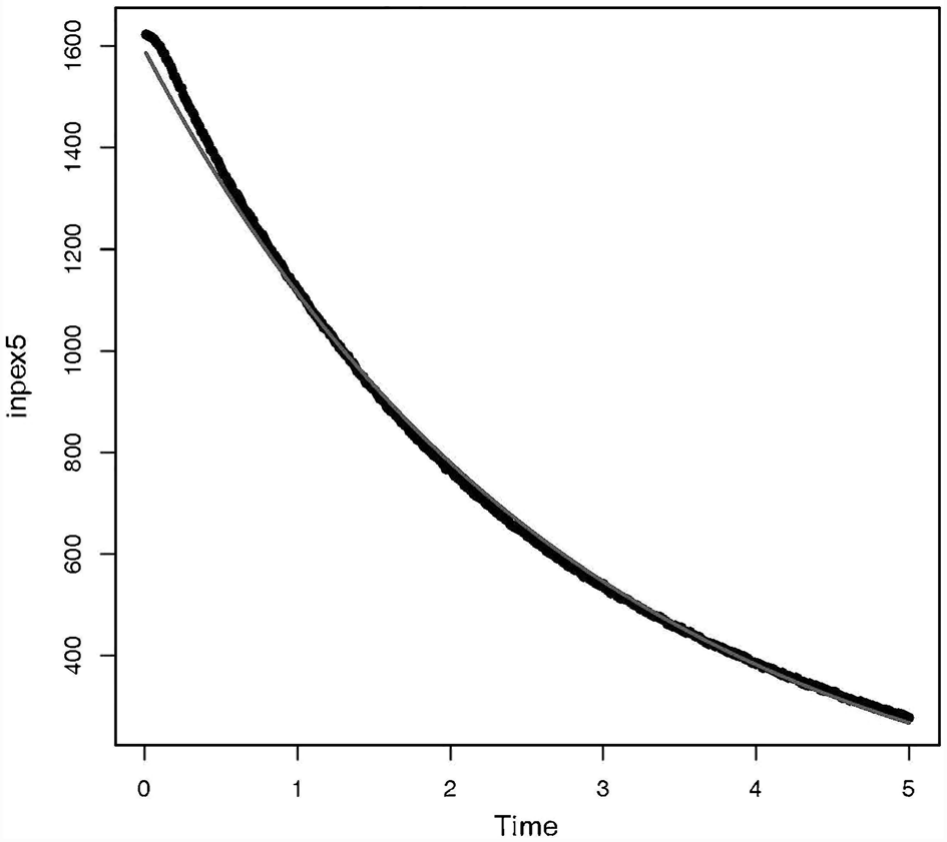
5 s-Syllectogram recorded on the blood of a healthy individual. The exponential curve (thin curve) fits the syllectogram with a r^2^coefficient of 0.998 (inpex5: intensity of the backscattered light, time in seconds).

The syllectogram, the curve describing the aggregation process, is, by convention, represented by the sum of two exponential functions, one for the fast formation of aggregates (rouleaux) and one for the formation of three-dimensional aggregates^11^. The two-by-two aggregation is at the origin of the fast formation of rouleaux described by the first exponential function. Therefore, we hypothesized that Model 2 (mono-exponential model) is the most appropriate for describing the initial EAK. Figure 5 shows an example of a syllectogram recorded during the first 5 s and the fitted exponential curve. To confirm our hypothesis, we tested the two models on a set of measurements.

#### Measurement of the concentration of isolated erythrocytes by light backscattering

Immediately after stirring the tube, isolated erythrocytes are randomly distributed in the blood. All parts of the erythrocytes can receive incident light (from the small side surface to the large disk shape surface). Since light is backscattered by red blood cell membranes, far more light is backscattered by the A form (isolated erythrocytes) than by the B form (two-by-two aggregates). At the very beginning of the aggregation process the decreasing intensity of backscattered light is only due to the decrease in number of isolated erythrocytes. Therefore, the mono-exponential model is very likely only valid for the initial phase of the EA process (e.g., the first 5 s). The variables delivered by the model are the time constant k of the exponential and the half-life (EAK5s for the 5 s segment): $\frac{Ln2}{k}$. The absolute intensities of backscattered light, including the initial value A0, are not meaningful. When two-by-two aggregates and more complicated rouleaux formations appear in significant numbers compared to isolated erythrocytes the curves become complex, and the mono-exponential model cannot be used. The mono-exponential fit of the data collected on longer periods (e.g., 10–20 s) gives lower fitting coefficients. The data recorded during 20 s (syllectogram) can be best fitted with the sum of two exponential functions : I(t) = I_f_ e^−t/kf^ + I_s_ e^−t/ks^ + I_0_ where I(t) is the intensity of the backscattered light, I_f_ the intensity of the light backscattered by fast forming aggregates, k_f_ the corresponding time constant, I_s_ the light backscattered by the slow forming aggregates, and k_s_ the corresponding time constant^11^. However, absolute numbers, including I_0_, become critical for the relative weights of the two exponential functions. The four constants of the model (two time-constants and two “initial” constants, I_f_ and I_s_) should be approximated at the beginning of the fitting algorithm^12^. For these reasons, we chose to limit our analysis to the early part of the EAK (formation of two-by-two aggregates).

### Validation studies

Table 2 summarizes the protocols of the three validation studies.

**Table 2:** Summary of the clinical study protocols.

Protocols	Subjects/setting	Questions
Study A	Healthy subjects/blood donation^a^	EAK description and modeling
Study B	Cardiology/ICU^b^	EAK in patients hospitalized in cardiology. Comparison with healthy subjects
Study C	Emergency department^c^	Stability of EAK measurement after blood sampling, intra-individual variability. Effect of hematocrit on EAK

^a^Etablissement Français du Sang, Besançon; ^b^Centre Hospitalier Sud-Francilien (CHSF), Corbeil-Essonnes, ^c^Lariboisière Hospital, Paris.

*Study A* was designed to measure EAK in healthy subjects, to compute the best mathematical model to fit the data and to establish reference data for comparison with data obtained in patients. Blood donors were chosen because the probability of disease is very low in this young, screened population. To confirm that the mono-exponential model is the best for the initial EAK we compared the fitting coefficients r^2^ of the two models on the data obtained in a group of healthy subjects. To test whether the quality of the commercial tubes can influence EAK measurement we collected the blood of one donor in two different tubes and measured EAK 30 times on each tube.

*Study B* was conducted in patients hospitalized in the intensive care unit of a cardiology department. This kind of patients is known to have EA with high critical shear stress (CCS)^13^. The main objectives of the study were to determine whether EAK can be measured with our technique in all these patients and whether we can discriminate patients with acute coronary syndrome from both healthy subjects (in comparison with Study A) and patients with other cardiovascular conditions.

The clinical protocols have been approved by the appropriate committees according to French law (CPP Ile-de-France VI, Groupe Hospitalier Pitié-Salpêtrière, 4 bâtiment de la Force, 47 boulevard de l’Hôpital 75,651 Paris cedex 13) and all subjects gave their informed consent.

**Figure 6:**
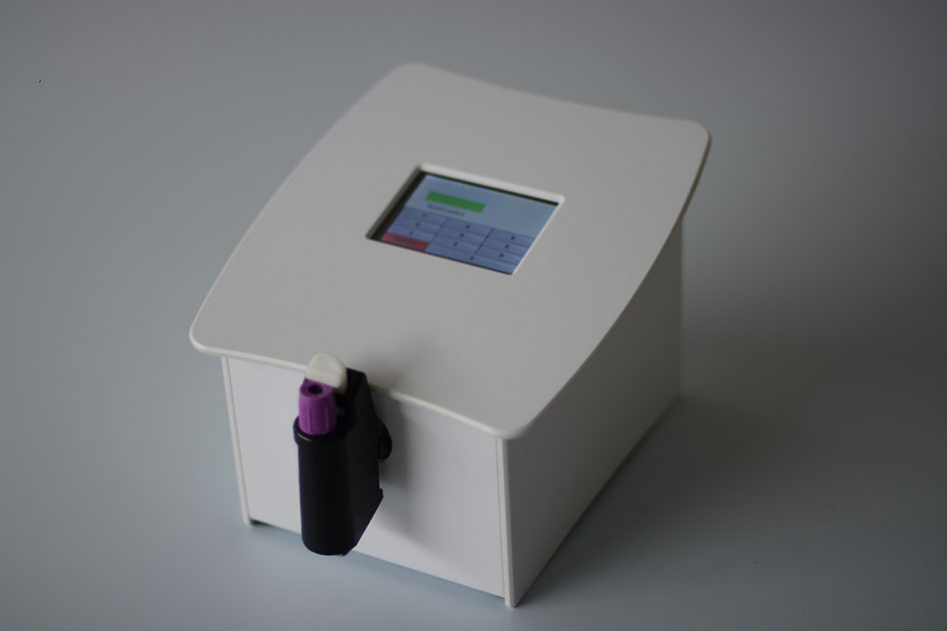
Point-of-care device with a EDTA collection tube (lavender cap) in the optical chamber, ready to be stirred.

*Study C* was designed as part of a larger study conducted among patients visiting an emergency department. The objective was to establish both EAK measurement stability one hour after blood sampling and its intra-individual variability. We also assessed the correlation between hematocrit and EAK. For this study, we used the advanced version of the device which can be used at the point of care in any clinical setting (Fig. 6).

### Data modelling and statistics

We used R software (R version 3.6.2, https://cran.r-project.org) and appropriate functions to fit the data. For each recorded set of data, the first 5 s segment was fitted (5 s model), and half-life was computed: ${\text{EAK5s}} = \frac{Ln2}{k}$, as well as the fitting coefficient r^2^ corresponding to the percentage of the variance explained by the model. We also fit the first 1.5 s, 3 s, and 10 s segments and computed the fitting coefficients (1.5 s, 3 s, and 10 s models). Therefore, we can determine which segment best fits the mono-exponential model.

For testing Model 1 we fit the inverse of the backscattered light intensity over time with a linear model and computed the r^2^ fitting coefficient. We compared the mean and median r^2^.

To find out whether EAK5s follows a normal distribution we used the Shapiro–Wilk test.

For group comparisons we used the Student’s t test (two-tailed).

We computed interindividual variation coefficients for each group of interest.

To explore the stability of EAK measurement over time we computed the variation coefficients and we used the Student’s test to compare measures taken both immediately after blood sampling and after one hour.

### Statement

All methods were carried out in accordance with relevant guidelines and regulations.

## Data Availability

Data from human subjects are managed in accordance with the study protocols and the French Law. The first author can provide technical support to duplicate the results.
